# Tyrosine Kinase Inhibitor Resistance Increased the Risk of Cerebral Radiation Necrosis After Stereotactic Radiosurgery in Brain Metastases of Non-small-Cell Lung Cancer: A Multi-Institutional Retrospective Case-Control Study

**DOI:** 10.3389/fonc.2020.00012

**Published:** 2020-02-11

**Authors:** Hongqing Zhuang, Liyuan Tao, Xin Wang, Siyu Shi, Zhiyong Yuan, Enmin Wang, Joe Y. Chang

**Affiliations:** ^1^Department of Radiation Oncology, Peking University Third Hospital, Beijing, China; ^2^Clinical Epidemiology Department, Peking University Third Hospital, Beijing, China; ^3^Department of Neurosurgery, Huashan Hospital, Fudan University, Shanghai, China; ^4^Stanford University School of Medicine, Stanford, CA, United States; ^5^Tianjin Key Laboratory of Cancer Prevention and Therapy, Department of Radiotherapy, National Clinical Research Center for Cancer, Tianjin Medical University Cancer Institute and Hospital, Tianjin, China; ^6^Division of Radiation Oncology, Department of Radiation Oncology, The University of Texas MD Anderson Cancer Center, Houston, TX, United States

**Keywords:** cerebral radiation necrosis, tyrosine kinase inhibitor, brain metastasis, epidermal growth factor receptor, stereotactic radiosurgery

## Abstract

**Objective:** This study aimed to investigate the relationship between the timing of stereotactic radiosurgery (SRS) intervention and the complications of cerebral radiation necrosis (CRN) in patients with brain metastases of lung adenocarcinoma who received tyrosine kinase inhibitor (TKI) treatment.

**Methods:** A total of 361 targets from 257 patients with brain oligometastases of lung adenocarcinoma who received CyberKnife treatment between 2010 and 2017 were retrospectively collected from three CyberKnife centers. The difference in brain necrosis between patients with or without TKI application was statistically counted. Logistic regression analysis was used to analyze the effect of applying TKI on the occurrence of CRN in patients and the effect of SRS before and after TKI resistance on CRN.

**Results:** The rate of CRN in the TKI group was significantly higher than that in the non-TKI group. The incidence of brain necrosis in patients undergoing SRS after drug resistance was significantly higher than that in patients undergoing SRS before drug resistance. Regression analysis showed that combination of TKI with SRS, and SRS after TKI resistance were important influencing factors for CRN.

**Conclusion:** Performing the SRS for brain metastases after TKI resistance worsened the occurrence of CRN of patients treated with TKI.

**Clinical Trial Registration:** Chinese clinical trial registry, http://www.chictr.org.cn/edit.aspx?pid=38395&htm=4, Registration number: ChiCTR1900022750.

## Introduction

Whether radiotherapy or targeted therapy should be performed first for brain metastases of non-small-cell lung cancer (NSCLC) has remained controversial. The recent research mainly focused on the survival (1–3). There were no reports concerning the increased occurrence of radiotherapy complications such as cerebral radiation necrosis (CRN), which further affected the quality of life in patients receiving stereotactic radiosurgery (SRS) after tyrosine kinase inhibitor (TKI) resistance. This study retrospectively analyzed the real-world case data on whether the TKI was applied when performing SRS, as well as the occurrence of CRN when performing SRS before and after TKI resistance, in patients with brain metastases of NSCLC from multiple centers. We investigated the TKI medication combined with SRS, and the occurrence of CRN and its impact on cognitive function and quality of life when performing SRS before and after drug resistance. This study was expected to provide evidence and reference for the treatment of such patients in clinical practice.

## Materials and Methods

### Case Data and Study Design

**Figure 1:**
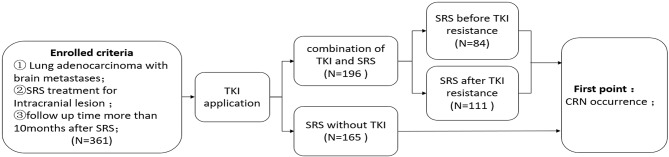
Study design.

A total of 361 lesions from 257 patients with brain metastases of lung adenocarcinoma were collected from the Peking University Third Hospital, Tianjin Cancer Hospital, Huashan Hospital affiliated to Fudan University between January 2010 and December 2017. The inclusion criteria were as follows: (1) intracranial metastases of lung adenocarcinoma (number of metastatic lesions ≤ 4); (2) SRS was performed for the intracranial metastases or whole-brain radiotherapy combined with SRS; and (3) the follow-up time after SRS was >10 months (4). The exclusion criteria were as follows: (1) the follow-up time was <10 months for patients with no brain necrosis after stereotactic radiotherapy; and (2) the local lesions could not be further characterized during their surgery after SRS. The study was approved by the Ethics Committee of the Peking University Third Hospital and completed under its supervision. It was in accordance with all the ethical requirements (Figure 1).

### Treatment and Examination Methods

All intracranial metastasis SRS was treated with stereotactic radiotherapy by CyberKnife (Table 1). The epidermal growth factor receptor (EGFR) mutations were detected by PCR, digital PCR, or second-generation sequencing; and the results were independently determined by more than three specialists. Patients with EGFR mutations (19 or 21 exon mutation) taking TKI medication were treated with the first generation of TKI (gefitinib, erlotinib, or icotinib). Patients without EGFR mutations were treated with chemotherapy-based comprehensive treatment. The patients in this study received routine enhanced magnetic resonance imaging (MRI) 2 months after treatment, and then the reexamination interval was determined according to the local lesions, which was at least four times in the first year and at least three times per year from the second year for patients with stable lesions, but the longest interval did not exceed 6 months. If there were intracranial symptoms, reexamination was immediately performed. CRN was diagnosed by comprehensive medical history, symptoms, and signs combined with dynamic observation of intracranial lesions and MRI examination. For patients with doubtful diagnosis of CRN by enhanced brain MRI, routine spectral analysis or PET-CT and other means were further performed for final diagnosis. Imaging diagnosis required three doctors to simultaneously diagnose the brain necrosis (4–6).

**Table 1:** Baseline data of patients.

	**Total**	**Patients with CRN**	**Patients without CRN**	***t*/χ^**2**^/*z***	***p***
Age^a^	59.58 ± 10.47	58.64 ± 11.02	59.80 ± 10.35	0.814	0.416
Gender^b^				0.313	0.576
Female	205 (56.8)	36 (17.6)	169 (82.4)		
Male	156 (43.2)	31 (19.9)	125 (80.1)		
TKI^b^				31.95	<0.001
Yes	195 (54.0)	57 (29.2)	138 (70.8)		
No	166 (46.0)	10 (6.0)	156 (94.0)		
Treatment volume^c^	2844.88 (871.58, 7866.17)	2771.79 (840.99, 9981.06)	2858.56 (883.70, 7243.09)	0.593	0.553
Fraction^c^	2 (1, 3)	2 (1, 3)	2 (1, 3)	1.936	0.053
Radiation dose (BED)^c^	70.40 (60.00, 75.90)	72.00 (60.00, 79.00)	70.40 (60.00, 75.90)	1.329	0.184

*BED, biological effective dose; CRN, cerebral radiation necrosis; TKI, tyrosine kinase inhibitor*.

a *The data are expressed as mean ± SD, and t-test was used for comparison*.

b *The data are expressed as n (%), and χ^2^ test was used for comparison*.

c *The data are expressed as median (p25, p75), and non-parametric test was used for comparison*.

### Statistical Analysis

Statistical analysis was performed using the SPSS 25.0 software. The measurement data in accordance with the normal distribution were expressed as mean ± standard deviation. The comparison between groups was performed by independent sample *t*-test. The measurement data that did not conform to the normal distribution was expressed as median (25% quantile, 75% quantile). The comparison between groups was performed using the non-parametric Mann-Whitney *U*-test. The enumeration data were expressed as the number of cases (percentage), and the comparison between groups was performed using the chi-square test. Logistic regression was used to analyze the effect of applying TKI on CRN in patients. Meanwhile, logistic regression was used to analyze the effect of performing SRS before and after drug resistance on difference in CRN in patients taking TKI medication. The statistically significant differences were accepted as bilateral *p* < 0.05 for all tests.

## Results

### The General Data of Cerebral Radiation Necrosis

For the 361 targets, a total of 67 targets (18.6%) had CRN. Among them, 57 (29.2%) cases had CRN in the TKI combination treatment group, and 10 (6.0%) cases had CRN in the non-TKI combination treatment group. The difference between the two groups was statistically significant (χ^2^ = 31.95, *p* < 0.001) (Table 1).

### Patients With Tyrosine Kinase Inhibitor Medication Were More Likely to Have Cerebral Radiation Necrosis Than Those Without Tyrosine Kinase Inhibitor

Logistic regression analysis was performed on factors such as age, gender, therapeutic dose, target volume, number of divisions, and whether patients used TKI. The results showed that TKI medication was an important prognostic factor for CRN. The risk of CRN in patients using TKI was six times higher than those who did not use drugs (Table 2).

**Table 2:** Regression analysis of TKI application on CRN in patients with SRS.

	**Univariate analysis**			**Multivariate analysis**		
	**OR**	**95% CI**	***p***	**OR**	**95% CI**	***p***
Age	0.990	0.965–1.015	0.415	0.993	0.967–1.019	0.585
Gender			0.576			0.748
Male	1.000			1.000		
Female	1.164	0.683–1.984		0.911	0.515–1.610	
Treatment volume	1.000	0.998–1.003	0.997	0.999	0.995–1.002	0.373
Fraction	1.230	0.957–1.581	0.106	1.217	0.883–1.676	0.231
Radiation dose	1.021	0.995–1.048	0.119	1.025	0.998–1.053	0.065
TKI			<0.001			<0.001
No	1.000			1.000		
Yes	6.443	3.168–13.105		6.193	2.986–12.845	

*TKI, tyrosine kinase inhibitor; CRN, cerebral radiation necrosis; SRS, stereotactic radiosurgery*.

### Comparison of Performing Stereotactic Radiosurgery Before and After Tyrosine Kinase Inhibitor Resistance

When TKI was applied in clinical practice, some patients did not undergo local radiotherapy owing to absence of symptoms after medical treatment. Radiotherapy was performed after the progression of drug resistance. However, other patients underwent brain radiotherapy at the initial period of drug use or before drug resistance. Statistical analysis of CRN occurrence before and after drug resistance in this group was performed by *t*-test, and the results showed a statistically significant difference between the two groups (*p* = 0.001). Regression analysis also showed that performing SRS after drug resistance was an important factor for the occurrence of CRN (Table 3). The incidence of CRN was significantly increased when the SRS was performed after drug resistance.

**Table 3:** Effects of SRS intervention time on CRN based on TKI therapy.

	**Univariate analysis**			**Multivariate analysis**		
	**OR**	**95% CI**	***p* value**	**OR**	**95% CI**	***p* value**
Age	0.986	0.959–1.014	0.313	0.970	0.940–1.001	0.057
Gender			0.839			0.815
Male	1.000			1.000		
Female	0.938	0.506–1.739		0.925	0.480–1.782	
Treatment volume	0.999	0.995–1.002	0.389	0.997	0.993–1.001	0.184
Fraction	1.075	0.801–1.441	0.631	1.415	0.962–2.081	0.078
Radiation dose	1.021	0.993–1.049	0.146	1.023	0.992–1.055	0.156
SRS			0.001			0.001
Before TKI resistance	1.000			1.000		
After TKI resistance	3.162	1.587–6.299		3.551	1.710–7.377	

*SRS, stereotactic radiosurgery; CRN, cerebral radiation necrosis; TKI, tyrosine kinase inhibitor*.

## Discussion

For brain metastases of EGFR-mutated NSCLC, TKI treatment and the timing of SRS during the TKI treatment had a direct impact on the occurrence of CRN. Performing SRS after TKI resistance increased the incidence of CRN. This was a useful analysis of the current clinical routine treatment mode of local radiotherapy after drug resistance for NSCLC patients with brain metastasis receiving TKI treatment.

Histological changes of intracranial tumor metastases after TKI treatment may be an important mechanism for the increase of CRN after SRS. First, in clinical practice, most of the intracranial metastases were small lesions and massive edema before TKI resistance, whereas most of the intracranial metastases were micro-edema or no edema after drug resistance, which was considered as a change in vascular status of the tumor. Preclinical studies have also shown that the blood vessels in the tumor tissues were significantly decreased after erlotinib treatment, as compared with pretreatment, and the tumor tissues showed significant ischemia and hypoxia. In contrast to pre-TKI resistance, tissue preparation for the occurrence of brain necrosis after SRS was indicated (7). Second, CRN occurred mainly owing to vascular injury (8–11). After TKI resistance, the vascular components in the tumor were significantly reduced (7, 12), and the local ischemia and hypoxia were more severe from the radiation-induced vascular injury, which was more likely to cause CRN.

This study had innovative significance for the clinical treatment mode of brain metastasis of EGFR-mutated NSCLC. The treatment mode of NSCLC with brain metastases in the TKI era was controversial. Professor Wu Yilong proposed a treatment mode of performing radiotherapy for local progression after TKI resistance (12, 13). However, studies published in the *Journal of Clinical Oncology* (*JCO*) (1) and *International Journal of Radiation Oncology, Biology, Physics* (2) suggested that the survival of patients with early radiotherapy was better in patients receiving TKI treatment. Nevertheless, all these studies did not mention the effects of performing radiotherapy after drug resistance or the long-term complications of radiotherapy. This study showed that it may worsen damage in the late period after drug resistance, which further affected the cognitive function and quality of life. Therefore, for patients with advanced EGFR-mutated NSCLC, if brain radiotherapy was not performed early on, it affected their survival, was more likely to cause CRN, and affected their quality of life. This was another exploration of the treatment mode for brain metastases of EGFR-mutated NSCLC patients.

This was a retrospective study. Given the difficulty of clinical research on radiotherapy in the TKI-targeted era and only few prospective studies on TKI combined with radiotherapy for brain metastasis (14), this retrospective data also had good reference value. Prospective studies can be conducted in the future to further confirm the results of this retrospective study (15).

## Conclusion

In summary, for brain metastases of NSCLC, TKI treatment and the timing of SRS intervention during TKI treatment had a direct impact on the occurrence of CRN. Performing the SRS after TKI resistance worsened the occurrence of CRN of patients treated with TKI. This study provided a good reference for the timing of TKI combined with radiotherapy in patients with brain metastases of EGFR-mutated NSCLC, clinical data for the development of treatment mode in such patients, and a useful exploration for improving the quality of life of the patients.

## Data Availability Statement

The datasets generated for this study are available on request to the corresponding author.

## Ethics Statement

The studies involving human participants were reviewed and approved by Peking University Third Hospital. The patients/participants provided their written informed consent to participate in this study. Written informed consent was obtained from the individual(s) for the publication of any potentially identifiable images or data included in this article.

## Author Contributions

All authors listed have made a substantial, direct and intellectual contribution to the work, and approved it for publication.

### Conflict of Interest

The authors declare that the research was conducted in the absence of any commercial or financial relationships that could be construed as a potential conflict of interest.
